# Dr. James K. King

**Published:** 1914-11

**Authors:** 


Dr. James K. King, P. & S. 1877, died at his home in Watkins, N. Y., September 20, aged 67. He was born and educated in Troy and took a partial course in medicine at the University of Michigan before attending the P. & S. After graduation, he studied in Edinburgh, Vienna and Berlin and was Assistant Master of the Rotunda Hospital of Dublin. On his return to the U. S., he became a member of the staff of the Clifton Springs Sanitarium where he remained till 1890, when he became one of the founders of the Glen Springs of Watkins and its medical director for 20 years. Following his retirement, he continued consulting practice. He was highly esteemed both professionally and socially.

